# Reversible sick sinus syndrome due to sinus node artery ischaemia in an elderly woman: a case report

**DOI:** 10.1093/ehjcr/ytaf660

**Published:** 2025-12-22

**Authors:** Yuji Kaneko, Tatsuro Hitsumoto, Masato Tada, Takuya Shimura, Akira Itoh

**Affiliations:** Division of Cardiology, HITO Medical Center, 788-1 Kamibun-cho, Shikokuchuo City, Ehime 799-0121, Japan; Division of Cardiology, HITO Medical Center, 788-1 Kamibun-cho, Shikokuchuo City, Ehime 799-0121, Japan; Division of Cardiology, HITO Medical Center, 788-1 Kamibun-cho, Shikokuchuo City, Ehime 799-0121, Japan; Division of Cardiology, HITO Medical Center, 788-1 Kamibun-cho, Shikokuchuo City, Ehime 799-0121, Japan; Division of Cardiology, HITO Medical Center, 788-1 Kamibun-cho, Shikokuchuo City, Ehime 799-0121, Japan

**Keywords:** Sick sinus syndrome, Pacemaker, Sinus node artery ischaemia, Coronary artery disease, Percutaneous coronary intervention, Case report

## Abstract

**Background:**

Sick sinus syndrome often necessitates permanent pacemaker implantation; however, when sinus node artery ischaemia is the cause, sinus node dysfunction may be reversible. While age-related fibrosis is generally considered the main mechanism, ischaemia should also be recognized as a potential aetiology.

**Case summary:**

An 80-year-old woman with breast cancer, hypertension, and diabetes mellitus presented with dyspnoea. Electrocardiography showed marked bradycardia at 38 beats per minute (b.p.m.) without ST-segment changes. Chest radiography revealed a cardiothoracic ratio of 61% with mild pulmonary congestion, and echocardiography showed preserved left ventricular ejection fraction (>60%) without wall motion abnormality. Although referred for pacemaker implantation, the acute onset of severe bradycardia together with multiple coronary risk factors prompted coronary angiography, which demonstrated 99% stenoses in the proximal right coronary and proximal left circumflex arteries, and 90% in the mid left anterior descending artery. Flow delay was noted in the sinus node branch of the right coronary artery. Percutaneous coronary intervention of the proximal right coronary artery promptly restored sinus rhythm during the procedure. She has since remained stable with a sinus rate of 70 b.p.m. for 1 year without pacing.

**Discussion:**

Despite no chest pain or ST changes, the acute presentation and multiple coronary risk factors suggested an ischaemic aetiology. Intermittent right coronary artery impairment, bradycardia-facilitated collateral flow, and repolarization abnormalities likely obscured typical ischaemic signs. This case highlights the importance of ischaemic evaluation before pacemaker implantation, even in elderly patients.

Learning points

- In elderly patients with sudden bradycardia, coronary ischaemia should be excluded before pacemaker implantation.
- Coronary revascularization may restore sinus node function and help avoid unnecessary device implantation and its associated complications.
- Collateral flow during bradycardia may mask typical ischaemic signs such as chest pain or ST changes.

## Introduction

Sick sinus syndrome (SSS) is a condition characterized by impaired sinus node function leading to bradycardia or sinus arrest and is one of the major indications for permanent pacemaker implantation in elderly patients. Although irreversible changes due to aging and fibrosis are generally considered the main causes, ischaemia of the sinus node branch (SNB) has also been reported as an aetiology.^1,2^ In such cases, relief of ischaemia may restore sinus node function, potentially allowing avoidance of pacemaker implantation.^3,4^

Most reports of sinus node dysfunction related to the SNB describe iatrogenic occlusion during percutaneous coronary intervention (PCI). By contrast, *de novo* ischaemia of the SNB causing severe bradycardia before any intervention is extremely rare. Recognizing this difference is important because it highlights the need for ischaemic evaluation in patients with unexplained bradycardia.

We report a case of severe bradycardia referred for pacemaker implantation. Coronary angiography (CAG) revealed multivessel disease, including severe proximal right coronary artery (pRCA) stenosis with delayed flow in the SNB. After PCI, sinus rhythm was restored, and permanent pacing was successfully avoided.

## Summary figure

**Figure:**
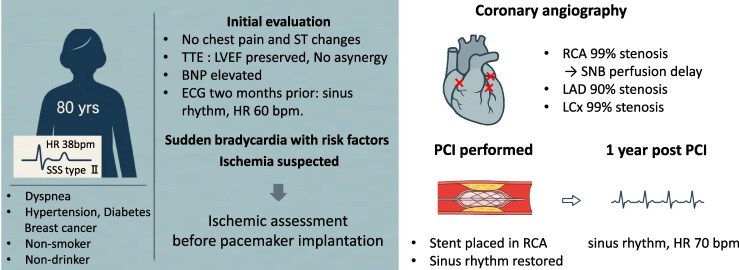


## Case presentation

An 80-year-old woman developed dyspnoea in late December 2024, which gradually limited her daily activities. In January 2025, she visited a local physician, where marked bradycardia at 38 beats per minute (b.p.m.) was observed, and she was referred to our department of cardiology for pacemaker implantation.

Her past medical history included breast cancer, hypertension, and diabetes mellitus. Medications included letrozole for breast cancer, angiotensin receptor blocker, DPP-4 inhibitor, and proton pump inhibitor. She had no history of taking drugs associated with bradycardia. She had no history of smoking or alcohol consumption, and no family history of cardiac disease.

On admission, she was alert with a body temperature of 36.4°C, blood pressure of 99/49 mmHg, heart rate of 38 b.p.m., respiratory rate of 12 breaths/min, and SpO₂ 95% on 2 L oxygen supplementation. Jugular venous distension was absent. Heart sounds were regular without murmurs, and breath sounds were clear. There was no peripheral coldness, with only mild pretibial oedema.

**Figure 1:**
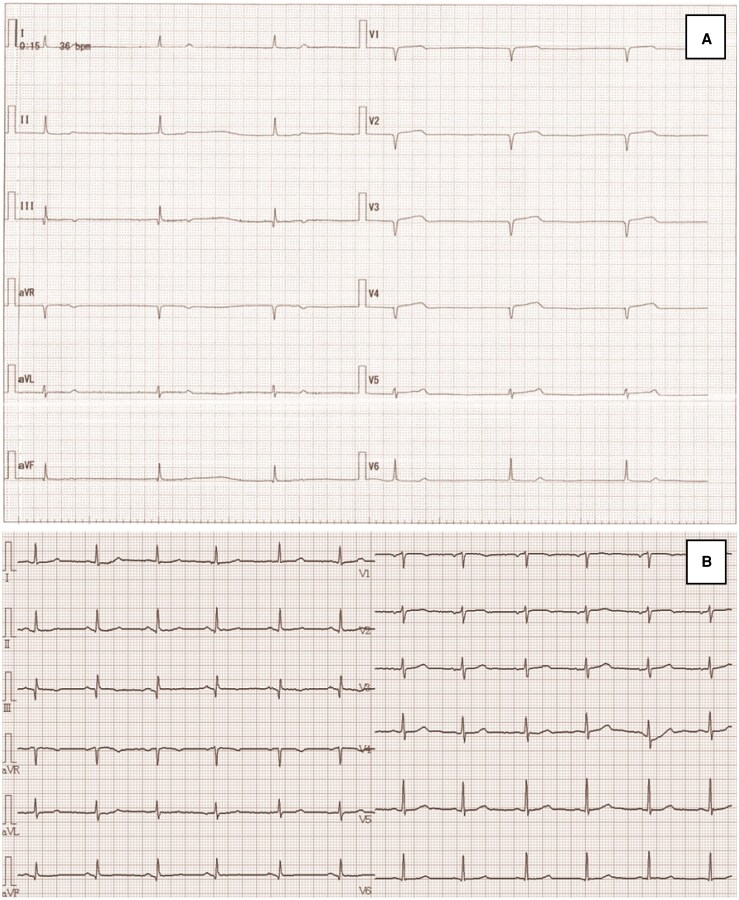
(*A*) Pre-percutaneous coronary intervention electrocardiography showing sinus arrest with a ventricular rate of 38 b.p.m. and a narrow QRS escape rhythm, without ST-segment changes. (*B*) Electrocardiography at 1-year follow-up showing stable sinus rhythm with a heart rate of 70 b.p.m.

Electrocardiography (ECG) revealed sinus arrest with a narrow QRS escape rhythm at 38 b.p.m., without ST-segment changes. There was no atrioventricular block or QT prolongation, and the escape rhythm had a narrow QRS configuration (*Figure 1A*). Chest radiography showed a cardiothoracic ratio of 61% with mild pulmonary congestion. Transthoracic echocardiography revealed preserved left ventricular ejection fraction (>60%) without regional wall motion abnormalities. Laboratory data showed elevated B-type natriuretic peptide (BNP) at 269.8 ng/mL, while cardiac enzymes were within normal ranges. Thyroid function and electrolytes were also within normal limits (*Table 1*).

**Table 1:** Laboratory data on admission

Parameter	Result	Reference range
RBC	385 × 10⁴/µL	380–480 × 10⁴/µL
Hb	11.4 g/dL	11.5–15.0 g/dL
Ht	34.7%	34–45%
WBC	8400/µL	3500–9000/µL
Plt	25.1 × 10⁴/µL	15–35 × 10⁴/µL
Blood glucose	139 mg/dL	70–109 mg/dL
HbA1c	5.5%	4.6–6.2%
AST	34 U/L	10–35 U/L
ALT	41 U/L	5–45 U/L
BUN	17.8 mg/dL	8–20 mg/dL
Creatinine	0.96 mg/dL	0.46–0.82 mg/dL
T-Cho	169 mg/dL	120–219 mg/dL
HDL-C	38 mg/dL	≥40 mg/dL
LDL-C	110 mg/dL	60–139 mg/dL
TG	100 mg/dL	30–149 mg/dL
TP	6.3 g/dL	6.5–8.2 g/dL
CK	94 U/L	40–150 U/L
CK-MB	2.6 ng/mL	<5 ng/mL
Na	141 mmol/L	138–146 mmol/L
K	4.2 mmol/L	3.6–4.8 mmol/L
Cl	107 mmol/L	101–108 mmol/L
Ca	9.4 mg/dL	8.5–10.2 mg/dL
C-reactive protein	0.29 mg/dL	<0.30 mg/dL
TSH	0.73 mIU/L	0.61–4.23 mIU/L
T3	2.65 pg/mL	1.68–3.67 pg/mL
T4	0.97 ng/dL	0.70–1.48 ng/dL
PT-INR	1.02	0.85–1.15
APTT	34.8 s	25–40 s
D-dimer	3.70 µg/mL	<1.0 µg/mL
BNP	269.8 ng/mL	<18.4 ng/mL

BNP was elevated at 269.8 ng/mL, whereas cardiac enzymes were within the normal range. Thyroid function tests and serum electrolytes were also within normal limits.

From these initial findings, there was no evidence of ischaemia, as chest pain, ST-segment changes, and ischaemic biomarkers were absent. An ECG obtained 2 months prior had documented sinus rhythm with a heart rate in the 60 b.p.m., suggesting relatively recent onset of bradycardia. Given her multiple coronary risk factors, including hypertension and diabetes mellitus, ischaemia was considered as a possible underlying cause. Therefore, instead of proceeding directly to pacemaker implantation, we decided to perform CAG.

**Figure 2:**
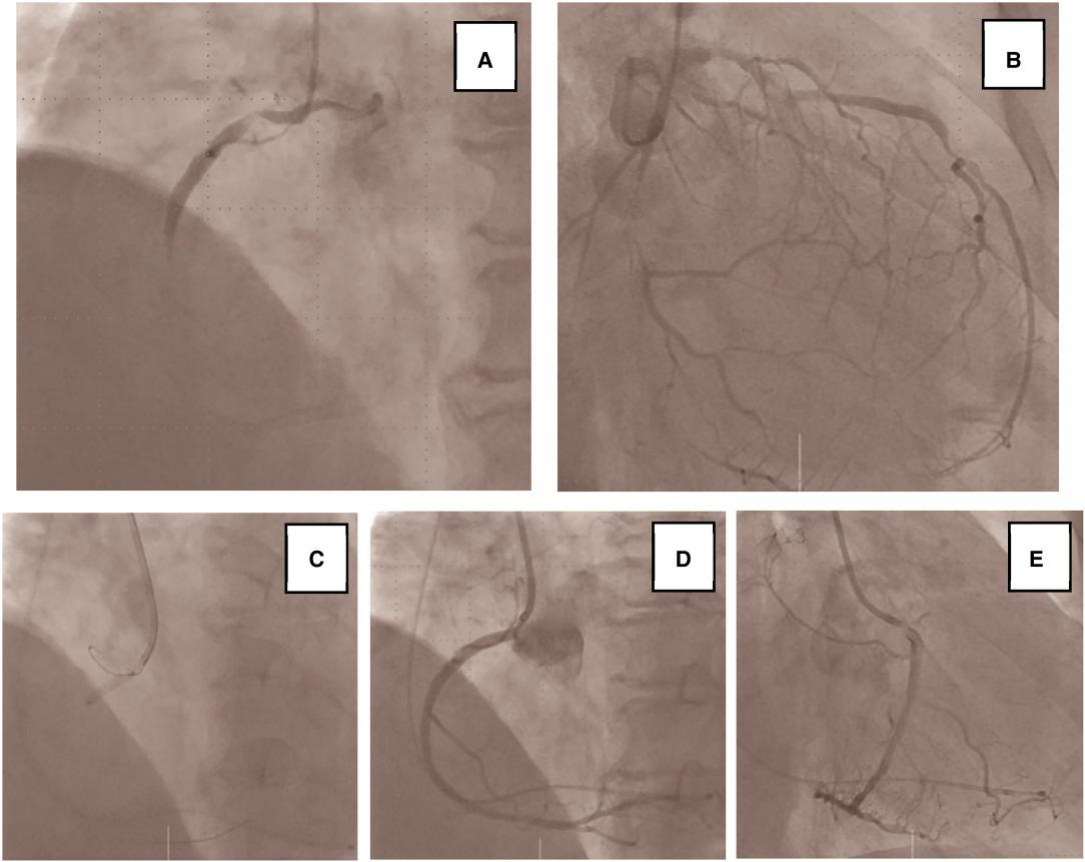
(*A*) Coronary angiography revealed 99% stenosis of proximal right coronary artery, with delayed flow in sinus node branch (Supplementary material online, *Video 2(A)*). (*B*) Coronary angiography revealed 99% stenosis of proximal left circumflex artery and 90% stenosis of mid left anterior descending artery. (*C*) Deployment of a Xience Skypoint stent (3.5 × 15 mm) in proximal right coronary artery. (*D, E*) Following stent implantation, coronary angiography showed restoration of TIMI III flow in right coronary artery territory, including sinus node branch (Supplementary material online, *Video (2E)*).

Coronary angiography revealed 99% stenosis in pRCA, 99% in the proximal left circumflex artery (pLCx), and 90% in the mid left anterior descending artery (mLAD). Flow delay was observed in the SNB arising from the RCA (*Figure 2A,B*). Based on these findings, we concluded that sinus node dysfunction was due to ischaemia of the SNB. The initially scheduled pacemaker implantation was cancelled, and temporary pacing was established while PCI was prioritized.

**Figure 3:**
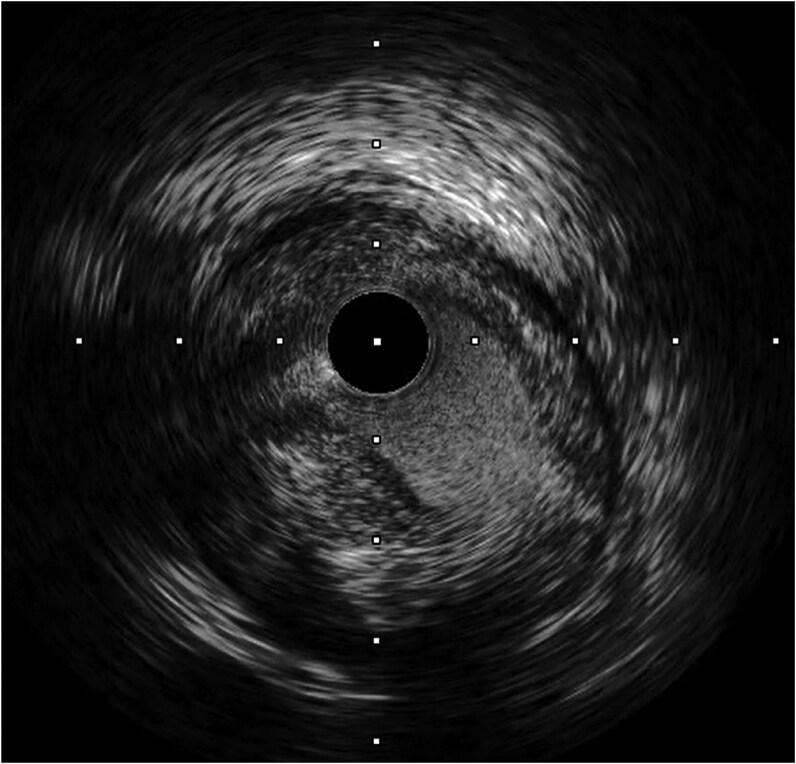
Intravascular ultrasound findings of proximal right coronary artery. Intravascular ultrasound demonstrated a mixed plaque with both fibrous and lipid components and the presence of thrombus, suggesting a relatively acute lesion responsible for sinus node artery ischaemia (Supplementary material online, *Video 3*).

Intravascular ultrasound of the pRCA demonstrated mixed plaque and thrombus, suggesting a relatively acute lesion (*Figure 3*). A Xience Skypoint stent (3.5 × 15 mm) was implanted in the pRCA (*Figure 2C*), achieving thrombolysis in myocardial infarction (TIMI) III flow in the RCA territory including the SNB (*Figure 2D, E*). Sinus rhythm with rates in the 50 s was restored during PCI. The temporary pacemaker was removed the following day, after which sinus rhythm was maintained and symptoms of heart failure improved.

**Figure 4:**
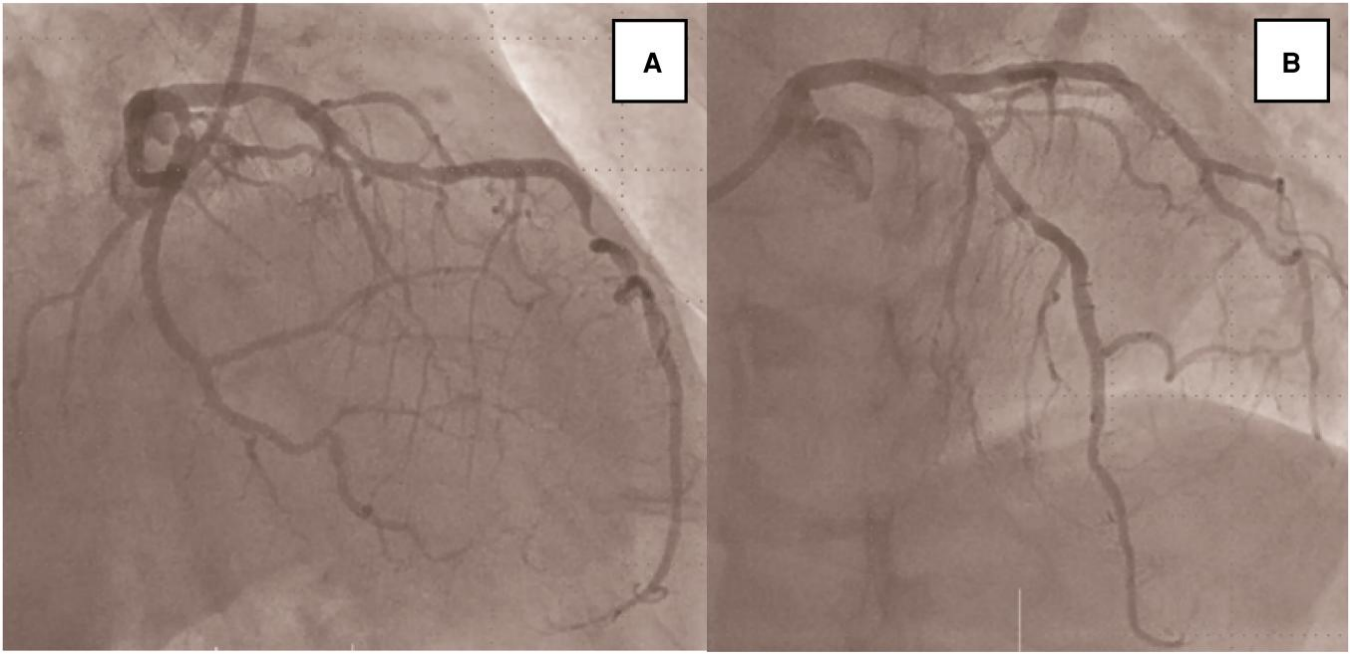
(*A*) Orsiro stent (2.25 × 30 mm) was deployed in proximal left circumflex artery. (*B*) Orsiro stent (2.5 × 18 mm) was deployed in mid left anterior descending artery.

Subsequent staged PCI achieved complete revascularization with additional Orsiro stents (2.25 × 30 mm in the pLCx and 2.5 × 18 mm in the mLAD (*Figure 4A, B*). At 1-year follow-up, she remained in stable sinus rhythm at 70 b.p.m. (*Figure 1B*). During this period, she experienced no recurrence of bradycardia or symptoms. Permanent pacemaker implantation was successfully avoided, confirming the reversibility of sinus node dysfunction in this case.

## Discussion

This case showed a reversible type of SSS caused by coronary artery disease. After revascularization, sinus node function completely recovered. In most elderly patients, SSS is due to irreversible fibrosis or aging. However, when ischaemia of SNB is present, restoring blood flow can improve sinus node activity.^1–4^ Although ischaemia is estimated to account for <15% of SSS cases,^5–7^ reports directly demonstrating SNB ischaemia as the cause are rare. This makes our case unique because the ischaemia was observed before any coronary intervention and was not iatrogenic.

In this case, there were no typical ischaemic findings such as chest pain, ST-segment changes, or elevation of cardiac enzymes. Several mechanisms may explain this. First, flow impairment of the RCA was intermittent, preventing progression to severe ischaemia. Second, collateral circulation from the LCx to the SNB may have limited ischaemic severity. Interestingly, bradycardia itself has been reported to promote the development of collateral circulation.^8,9^ Gloekler *et al*.^10^ reported that ivabradine-induced heart rate reduction enhanced collateral function in patients with stable coronary artery disease. This adaptive mechanism may have masked ischaemic signs and contributed to the clinically silent presentation. Previous studies also reported that even when the SNB is occluded during PCI, nearly half of patients remain asymptomatic.^6^ Additionally, under a ventricular escape rhythm, abnormal repolarization can hide ischaemia-related ST changes, making diagnosis even more difficult.

The important implication of this case is that bradycardia is not always caused by irreversible disease; ischaemia may represent a reversible aetiology. In particular, when elderly patients present with acute-onset bradycardia and multiple coronary risk factors, ischaemia should be strongly suspected.

Revascularization should be performed before permanent pacemaker implantation, and sinus node recovery should be observed. This strategy avoids unnecessary device implantation and related complications, improving long-term quality of life. This approach is consistent with the ESC pacing guidelines, which recommend excluding reversible causes such as ischaemia before device implantation.^11^

This case highlights that even in patients referred for pacemaker implantation, underlying coronary artery disease may be present. For similar cases in the future, rather than proceeding directly to device implantation, thorough ischaemic evaluation is of great clinical importance. This case serves as an educational reminder that careful diagnostic reasoning, guided by clinical and angiographic evidence, is crucial even in apparently straightforward cases of bradycardia.

## Conclusion

We reported an elderly woman with severe bradycardia caused by ischaemia of the SNB. After PCI, sinus rhythm recovered, and permanent pacemaker implantation was successfully avoided. This case demonstrates that even when typical ischaemic signs are absent, ischaemia should be considered as a reversible cause of bradycardia. Careful ischaemic evaluation before pacemaker implantation can prevent unnecessary procedures and improve patient outcomes.

## Supplementary Material

ytaf660_Supplementary_Data

## Data Availability

The data underlying this article are available in the article.

## Lead author biography

Yuji Kaneko, MD, is a first-year resident at HITO Hospital, Shikoku-Chūō City, Japan.
